# When Helpers Go Above and Beyond: Development and Characterization of Cytotoxic CD4^+^ T Cells

**DOI:** 10.3389/fimmu.2022.951900

**Published:** 2022-07-12

**Authors:** Cindy Hoeks, Gayel Duran, Niels Hellings, Bieke Broux

**Affiliations:** ^1^ Neuro Immune Connections & Repair Lab, Department of Immunology and Infection, Biomedical Research Institute, Hasselt University, Hasselt, Belgium; ^2^ University MS Center (UMSC), Hasselt, Belgium

**Keywords:** CD4 CTL, cytotoxic T cell, Th differentiation, migration, CD4 cytotoxic cells

## Abstract

Once regarded as an experimental artefact, cytotoxic CD4^+^ T cells (CD4 CTL) are presently recognized as a biologically relevant T cell subset with important functions in anti-viral, anti-tumor, and autoimmune responses. Despite the potentially large impact on their micro-environment, the absolute cell counts of CD4 CTL within the peripheral circulation are relatively low. With the rise of single cell analysis techniques, detection of these cells is greatly facilitated. This led to a renewed appraisal of CD4 CTL and an increased insight into their heterogeneous nature and ontogeny. In this review, we summarize the developmental path from naïve CD4^+^ T cells to terminally differentiated CD4 CTL, and present markers that can be used to detect or isolate CD4 CTL and their precursors. Subsets of CD4 CTL and their divergent functionalities are discussed. Finally, the importance of local cues as triggers for CD4 CTL differentiation is debated, posing the question whether CD4 CTL develop in the periphery and migrate to site of inflammation when called for, or that circulating CD4 CTL reflect cells that returned to the circulation following differentiation at the local inflammatory site they previously migrated to. Even though much remains to be learned about this intriguing T cell subset, it is clear that CD4 CTL represent interesting therapeutic targets for several pathologies.

## Introduction

T cells are a crucial part of the immune system due to their capability to provide long term protection against pathogens. Each different type of T cells has its own function within the development and maintenance of this long-lasting immunity. Classically, the T cell population is subdivided into helper and cytotoxic cells, bearing the CD4 and CD8 co-receptors respectively next to the CD3 T cell receptor complex (1). It is now becoming increasingly clear that there are exceptions to this rule, and that CD4^+^ T helper cells can also bear cytotoxic traits. In this review, we will focus on the development and phenotype of this rare population known as cytotoxic CD4^+^ T cells (CD4 CTL), that shares characteristics of both helper and cytotoxic T cells.

## T Cell Development

### Development of Thymocytes Into Mature T Cells

T cell development starts when bone marrow-derived lymphocyte precursor cells enter the thymus. These thymocytes go through several stages before differentiating into CD4^+^ or CD8^+^ single positive (SP) T cells. First, double negative (DN) thymocytes, which lack expression of both CD4 and CD8 co-receptors, expand in number under the influence of IL-7. Then, after a series of re-arrangements of T cell receptor (TCR) genes and assembly of the TCR complex, DN cells either die by apoptosis, due to unsuccessful re-arrangement, or become double positive (DP) cells expressing both CD4 and CD8, next to a functional TCR. Next, DP thymocytes will undergo the process of positive selection, which is dependent on engagement with antigen presenting MHC molecules, and failure to do so leads to apoptosis (2). DP cells with a moderate affinity for MHC molecules will survive this selection process and become single positive cells, while cells with strong TCR binding to antigen presenting MHCs, reflective of self-antigen recognition, undergo negative selection, also resulting in cell death by apoptosis. The lineage commitment of single positive cells depends on their recognition of class I or class II MHC molecules, committing them to CD8 or CD4, respectively (3, 4). The transcription factors ThPOK and RUNX3 are regarded as the master regulators of this T cell lineage commitment. Induction of ThPOK in MHCII-signaled thymocytes is both necessary and sufficient for the CD4 helper lineage commitment. Similarly, RUNX3 induction in MHCI-signaled thymocytes establishes a cytotoxic program in the CD8-committed thymocytes (5, 6).

### Naïve CD4+ T Cells can Differentiate Into Various Memory Th Subsets

After maturation in the thymus, naïve CD4^+^ and CD8^+^ T cells enter the systemic circulation, where homing to specific tissues is primarily driven by chemokines and their corresponding receptors (7–9). These molecules co-ordinate cell migration into secondary lymphoid organs, where T cells interact with antigen-presenting dendritic cells (DCs) (10, 11). The differentiation status of CD4^+^ T cells determines their pattern of migration. Naïve cells express C-C Chemokine receptor 7 (CCR7) and CD62L, which direct homing to the T cell zones of the secondary lymphoid organs (12, 13). Recognition of and binding to their cognate antigen leads to activation *via* the TCR, co-stimulated by CD28 and CD4. After activation, CD45, a leukocyte common antigen present on the surface of lymphocytes, changes from its long isoform CD45RA to the short isoform CD45RO which is characteristic of memory T cells (14). Memory T cells can further be subdivided into central memory (T_CM_; CD62L^+^CCR7^+^CD45RA^-^CD45RO^+^), effector memory (T_EM_; CD62L^-^CCR7^-^CD45RA^-^CD45RO^+^), and effector memory re-expressing CD45RA (TEMRA; CD62L^-^CCR7^-^CD45RA^+^CD45RO^+/-^) T cells (15–17). Unlike naïve T cells and T_CM_ cells, which traffic towards lymphoid tissues, T_EM_ cells migrate to sites of inflammation to aid in the local immune response (1). Following antigen recognition and activation, CD4^+^ T cells can differentiate into different subsets of helper cells depending on TCR signal strength and local cues. These helper subsets are classically categorized as Th1, Th2, and Th17, depending on the cytokines they produce. Th cells under control of the transcriptional regulator T-bet, differentiate into Th1 cells and secrete IFN-γ and TNF-α. Th2 cells are characterized by expression of GATA3 and production of IL-4 and IL-5. Polarization towards Th17, distinguished by IL-17 secretion, is regulated by RORγt (18). Complexity of the CD4 T helper classification is ever-increasing as several other CD4^+^ T cell phenotypes are being identified. The terminology defining Th1/Th2/Th17 subsets therefore continues to be a subject of discussion. However, addressing all of these subsets and their defining characteristics is beyond the scope of this review (see (19) for a recent review on this topic).

### Terminal Differentiation Into CD4 CTL

It has been described that a portion of memory Th cells gradually loses expression of the co-stimulatory receptor CD28, with CD4^+^CD28^-^ cells exhibiting a distinct cytotoxic phenotype (20). These cells are now recognized as cytotoxic CD4^+^ T cells (CD4 CTL). At first, the development of these CD4 CTL was regarded as an artifact of *in vitro* cell culture (21), but this view changed when circulating CD4^+^perforin^+^ T cells were identified (22). Expansion of CD4^+^perforin^+^ cells was found in patients with chronic viral infections, and loss of the co-stimulatory molecule CD28 was associated with perforin expression (22). Upon determining that CD4 CTL commonly express oligoclonal antigen receptors with restricted antigen diversity, it was suggested that repeated antigenic stimulation leads to expansion of CD4 CTL (23, 24), for instance by a latent viral infection or by a common dietary antigen. Loss of co-stimulatory receptor CD28 however apparently does not result in decreased antigen avidity of CD4 CTL, since CD4 CTL are still capable of responding to specific antigens or anti-CD3 triggering *in vitro* (25–27). It should be noted that addition of feeder cells to *in vitro* cultures amends CD4 CTL proliferation (26), indicating that some alternative co-stimulatory signal might still be required to enhance their activation. Coupled with the observation that CD4 CTL considerably upregulate their cytotoxic potential when encountering pro-inflammatory cytokines like IL-2 and IL-15 (28, 29), it was proposed that these cells develop as an additional line of defense bridging innate and adaptive immunity. Primed by the chronic activation provided by the persistent presence of their cognate antigen, CD4 CTL can exert potent effector functions in a pro-inflammatory environment. This increased cytokine production by CD4 CTL can also, non-specifically, enhance the immune response directed to other antigens *via* bystander activation of CD4 CTL (30, 31). While CD4 CTL are now recognized to be of vital importance in anti-viral and anti-tumor immune responses (32), they can also contribute to chronic inflammatory pathologies (25, 33, 34). For instance, in patients suffering from multiple sclerosis (MS), expansion of CD4 CTL was correlated with disease severity and rate of disease progression following diagnosis (34). Depending on the context, either harnessing the full potential of CD4 CTLs, or preventing their development, holds great promise for therapeutic purposes. Therefore, it is essential to understand how CD4^+^ T cells develop into terminally differentiated CD4 CTL, and which markers distinguish precursor from effector CD4 CTL.

## Development of CD4 CTL

### Transcription Factors Regulating the Development of CD4 CTL in Mice

The first clues regarding the pathways involved in development of CD4 CTL came from studies using murine models (30). CD4 CTL development was found to be regulated by the transcription factors ThPOK and RUNX3. When RUNX3 is activated and represses ThPOK, a cytotoxic profile is established in CD4^+^ T cells similar to that of CD8^+^ effector cells. This process is additionally regulated by the Th1-associated transcription factor T-bet or by Eomes (29, 35). This is in accordance with other studies showing the capacity of T-bet and Eomes to induce both perforin- and Fas/FasL-mediated cytotoxicity in murine CD4^+^ cell lines (36, 37). T-bet alone is, however, not always sufficient to promote expression of cytotoxicity-related genes. Following influenza virus infection, CD4 CTL development at the site of infection required expression of the transcriptional repressor Blimp-1 in addition to upregulation of T-bet (38). In transgenic mice with B cells expressing the EBV oncoprotein latent membrane protein 1, CD4 CTL development was induced by activation of co-stimulatory receptors CD27 and OX40. This led to induction of Eomes or T-bet. Eomes activation in turn led to expression of granzyme B (GrB), while induction of T-bet was not necessary for GrB expression (39).

### Transcription Factors Regulating the Development of CD4 CTL in Humans

Human CD4 CTL development appears to differ slightly from their murine counterparts. A recent study using cytomegalovirus (CMV) as a model to induce CD4 CTL, showed that ThPOK was not downregulated in human CD4 CTL *ex vivo*, while these cells did express cytotoxic molecules. However, knockdown of ThPOK in naïve CD4^+^ T cells followed by *in vitro* stimulation to induce CD4 CTL development, enhanced the cytotoxic capacity of CD4 CTL. The transcriptional program of CD4 CTL was highly similar to that of CD8 CTL, with CD4 CTL expressing GrB, granzyme K (GrK), granulysin, and perforin. This process was regulated by RUNX3 and T-bet, while Eomes was found to be redundant for GrB or perforin expression. Interestingly, CMV-induced CD4 CTL developed exclusively from Th1-polarized cells, and the authors proposed that the initial steps of Th1 cell differentiation provide the required epigenetic and transcriptional signals necessary for expression of perforin (40). This is in contrast with other papers claiming that CD4 CTL should be regarded as separate Th subset and not merely terminally differentiated Th1 cells (41, 42). Given these discrepancies, it appears that acquisition of cytotoxic features is regulated *via* more than one pathway. Involvement of transcription factors like T-bet and Eomes might vary depending on cell polarization and environmental factors. Even different epitopes derived from the same virus but implicated in different phases of infection, in this case CMV, induced differential expression of T-bet and Eomes in CMV-specific CD4^+^ T cells. CD4^+^T-bet^Hi^Eomes^Hi/Lo^ cells were found to co-express IFN-γ and TNF-α upon CMV stimulation. Moreover, perforin, but not GrB, was increased in these highly differentiated double-positive cells compared to less differentiated cells expressing TNF-α only (43). This is in line with the view that perforin-expressing CD4^+^ T cells are Th1 polarized. Unfortunately, functional differences between CD4^+^T-bet^Hi^Eomes^Hi^ and CD4^+^T-bet^Hi^Eomes^Lo^ cells were not investigated, which would have revealed whether or not Eomes is redundant for perforin expression. In contrast, recent studies have shown that expression of Eomes in Th17-derived Th1 cells, CD4^+^CD27- CTL, and Tr1 cells induced expression of perforin and granzymes, and degranulation upon stimulation (44–46). However, it should be noted that these data are mostly derived from genetic overexpression studies, which could exaggerate the effect of Eomes *in vivo*. Interestingly, dendritic cell-derived IL-27 restricted development of CD4 CTL after CMV infection by downregulating T-bet, but not Eomes (47). Furthermore, the transcription factor Hobit, a homolog of Blimp-1, has been identified to regulate development of CD4 CTL after CMV infection, most likely mediated by T-bet following viral clearance (48). It appears that T-bet is one of the master regulators of human CD4 CTL development, at least in Th1-skewed precursors. However, acquisition of cytotoxic features, alongside induction of IFN-γ production, in non-Th1 precursors still seems possible, and likely occurs through induction of Eomes.

### Identifying Precursors of CD4 CTL

Now that the effector functions of CD4 CTL become increasingly clear, it is of interest to predict which cells reach this stage of terminal differentiation. A recent study showed that GrB production was induced in the majority of the total CD4^+^ population *in vitro* after short-term stimulation with a superantigen (49), suggesting that most, if not all CD4^+^ T cells have the potential to become cytotoxic given the right circumstances. However, this is not reflected in the circulating pool of CD4 CTL, which even in supercentenarians (≥110 years of age) is limited to about 25% of the total CD4^+^ population (50). A recent study identified a subset of memory T cells expressing low levels of KLRG1 and high levels of CD127 (IL-7R) as precursor CD4 CTL, as these markers were respectively up- and downregulated in effector CD4 CTL cells and shared TCR clonotypes were identified between the two cell populations (51). However, the usefulness of this combination of markers can be debated, as CD127 is expressed abundantly on CD4^+^ T cells. A more specific precursor marker was found in the class I-restricted T cell-associated molecule (CRTAM), as CRTAM expressed on murine naïve CD4^+^ T cells effectively predicted acquisition of cytotoxic features (52). These findings however need more validation, especially if they are to be used as therapeutic targets.

## Classification of CD4 CTL

Since their discovery, several studies have focused on describing phenotypes of CD4 CTL that arise as a consequence of aging or various pathologies (see (30, 33, 34, 41, 53–58) for most recent reviews). Several different markers have been used to define subsets, complicating the comparison of CD4 CTL across studies. Furthermore, negative markers such as loss of CD28 expression, or very general cytotoxic markers shared by other cell types are less useful from a practical perspective. Even though there is a clear need for a specific positive (surface) marker identifying CD4 CTL, the heterogeneous nature and plasticity of CD4^+^ T cells challenges its discovery. Alternatively, markers that discriminate pathogenic from protective CD4 CTL can be highly valuable for clinical use. Rapidly evolving techniques like single cell multi-omics analysis and high parameter flow- and mass cytometry now allow screening of CD4^+^ T cell subsets for distinguishing markers that might otherwise be overlooked.

### Markers Associated With Cellular Signaling

The four classical CD4^+^ differentiation stages are commonly identified based on CCR7 and CD45RA expression as discussed above, with effector memory (CCR7-) T cells that re-express CD45RA (TEMRA) being regarded as terminally differentiated cells. It has become apparent that the TEMRA subset is a very heterogeneous population and that not all TEMRA cells display cytotoxic properties (16, 50, 51, 59). Similarly, not all CD4 CTL re-express CD45RA (59). Analysis at the single cell level recently provided more insight into the heterogeneous nature of these subsets, identifying KLRB1, KLRG1, KLRF1, and GPR56 as markers to distinguish between pro-inflammatory and exhausted memory cells based on their relative level of cytokines produced. Cytokine production correlated better with KLRG1 and GPR56 expression than with conventional EM/EMRA classification (60). Another study recently described CD29 (integrin β1) as marker for CD4 CTL, with CD29^hi^CD4^+^ T cells displaying a cytotoxic gene profile that was to some extent reflected in *ex vivo* protein expression (61). Although the authors showed that CD4^+^ cells expressing cytotoxic molecules co-expressed CD29, the specificity of CD29 as marker for CD4 CTL was lacking. As only a small fraction of the CD29^hi^ cells expressed cytotoxic molecules on the protein level, the authors concluded that CD29 expression can be used to enrich for CD4 CTL (61). However, it can be debated that other markers better correlating with cytotoxicity, such as loss of CD28 expression, are more useful in this context.

### Markers Associated With NK Cells

Molecules expressed by NK cells are also considered as candidate markers for CD4 CTL. On CD4^+^CD28^-^ T cells, expression of CD57, KLRG1, KIR2DL1-2, CD244, NKG2D, NKG2C, and KIR2DS1/3/5 was found to be increased compared to CD4^+^CD28^+^ T cells, although expression levels varied greatly between donors (62). Of these NK-receptors, the activating receptor NKG2D is most studied in the context of CD4 CTL. NKG2D is expressed on a subset of CD4^+^ T cells (63), and was found to be a marker for pathogenic Th1 and Th17 cells co-producing IFN-γ, IL-17, and GM-CSF in a murine model for rheumatoid arthritis. Although similar in cytokine expression, these Th subsets had distinct cytotoxic profiles, since NKG2D^+^ Th1 cells showed increased gene expression of granzyme A (GrA) and GrB, while NKG2D^+^ Th17 cells upregulated perforin (64). In influenza A virus (IAV) infected mice, NKG2C/E expression was found on tissue resident CD4 CTL cells. NKG2C/E expression in this model correlated with Blimp-1 expression, while these cells lacked Eomes expression (65). The NK-cell maturation marker CD57 was found to be expressed on CMV-specific terminally differentiated CD4^+^ T cells (43), but since not all CD4^+^perforin^+^ were CD57^+^ this cannot be regarded a truly specific CD4 CTL marker. Overall, expression of NK-associated markers appears to be very heterogeneous throughout the CD4 CTL population, with their shared expression on other immune cell subsets such as NK cells and CD8 cells further complicating their use as therapeutic targets.

### Markers Associated With Cytotoxicity

CD4 CTL are described to express several cytotoxic markers typically associated with effector CD8^+^ T cells and NK cells, such as GrA, GrB, GrK, granulysin, and perforin. While several types of antigenic stimulation are known to induce development of CD4 CTL (41, 54, 57), it appears that not all antigens result in the same cytotoxic profile. For instance, CD4 CTL from CMV seropositive donors arise from Th1 precursor cells and utilize the perforin-pathway to exert their cytotoxic properties (40, 41). In contrast, dengue-virus (DENV) specific CD4 CTL lack expression of Th1-associated chemokine receptors and show Fas-FasL mediated killing (57). Interestingly, allograft rejection induced by CD4 CTL appears to involve a combination of perforin and Fas-FasL-mediated cytotoxicity (66). Regarding CD4 CTL in EBV there are some conflicting findings in literature. One study reported that *ex vivo* cytotoxicity was limited to EBV-specific CD4^+^ T cells in patients with primary EBV infection (infectious mononucleosis; IM) and was not detected in EBV seropositive healthy donors (67), in contrast to other chronic viral infections like CMV (59). However, a recent study on immune responses targeting EBV capsid proteins found that perforin and GrB expression in EBV-specific CD4^+^ T cells was maintained in the latent phase of EBV infection (68). In IM patients, the acutely generated EBV^+^ CD4 CTL had a Th1-like profile and expressed both GrB and perforin. Although expression of other cytotoxic markers like fractalkine receptor (CX3CR1), Eomes, and Hobit were increased within this population, none of these markers defined all EBV^+^ CD4 CTL. This suggests functional differences between acutely generated CD4 CTL and CD4 CTL that develop upon chronic stimulation, most likely arising from differences in differentiation status (respectively early- versus late-differentiated) when acquiring cytotoxic features (67).

## CD4 CTL: More Than Just One Subset

Even though expansion of CD4 CTL is in general limited in absolute numbers (at least when measured in peripheral circulation), recent studies at the single cell level have shown that CD4 CTL are a heterogeneous population. As discussed above, a study using CD4^+^ TEMRA cells isolated from donors previously infected with Dengue virus identified four separate clusters based on KLRG1 and IL-7 receptor (CD127) expression (51). Two of these TEMRA clusters were enriched for cytotoxicity-related genes, and differences in expression between these two clusters indicated that one cluster might preferentially utilize the Fas-FasL pathway and the other cluster the perforin pathway to exert their cytotoxic function. The differentiation status of these clusters was found to range from CM to TEMRA based on their expression profile of markers like CD27, CD28, LTB, and JUNB (51). In CMV infection, two distinct subsets of CD4 CTL were identified that shared a large number of TCR repertoires and both expressed GrB and perforin, but varied in their expression of chemokines (CCL5 vs CCL3 and CCL4) (69). Additionally, as mentioned before, the phenotype of CMV-reactive CD4 CTL can also vary for different CMV epitopes (43). This might be explained by differences in antigen presentation (59), supporting the notion that diverse antigens trigger diverse CD4 CTL phenotypes.

## Role of the Micro-Environment in CD4 CTL Development

Several studies report that CD4 CTL can be detected in the circulation as well as at the site of inflammation (30). Thus it can be questioned whether these are similar subsets of CD4 CTL, and if so, what happens first. Do CD4^+^ T cells develop into CD4 CTL in the periphery prior to migration? Or, do these cells migrate first and develop into CD4 CTL at the inflamed tissue, and return to the circulation once the local inflammation is resolved, similarly to effector memory T cells? Most evidence points to the second option. CD4 CTL generation requires repeated antigen presentation and inflammatory signaling, which can both be found near or at the site of inflammation (54). Pro-inflammatory cytokines furthermore contribute to accelerated loss of CD28 (58). Interestingly, next to the type of cytokines present, the amount of antigen presented determines the functionality of CD4 CTL, as it was reported that low amounts of antigen led to the highest levels of cytotoxicity (70). Such high avidity CTL can recognize limited amounts of antigen and are therefore very effective at clearing viral infections (54). Furthermore, several studies report the presence of CD4 CTL in inflamed tissues. In atherosclerotic plaques, increased expression of OX40L and 4-1BBL was found, and CD4^+^CD28^-^ cells present within these plaques had a highly activated phenotype. This suggests that local re-activation of (precursor) CD4^+^ CTL in peripheral tissues amplifies inflammation in the target organ, possibly leading to breakage of self-tolerance and therefore induction of autoimmune responses (33, 71). Similarly, within the population of intra-epithelial lymphocytes in the gut, CD4 CTL are found, which appear to be derived from intestinal Tregs and are induced to become CD4 CTL by retinoic acid and TGFβ signaling (42). In IAV infected mice, tissue resident CD4 CTL cells were identified that were absent in peripheral circulation. The authors hypothesized that CD4 CTL require some form of additional differentiation cues that most likely are present in the micro-environment of the infected tissue (65). A recent study performing single-cell RNA sequencing of blood and cerebrospinal fluid (CSF) samples from HC and MS donors revealed that the CSF of MS patients is enriched with CD4 CTL. Four subsets of CD4 CTL were identified that were CD27^-^CD28^-^, but differed in their expression of cytotoxic markers like perforin, GrB, and granulysin, but also CX3CR1, Eomes, T-bet, and Hobit (72). This heterogeneity supports the idea that precursor CD4 CTL migrate to the site of inflammation, where they further develop into effective CD4 CTL. However, infiltration into tissues is promoted by chemokine receptors and effector molecules expressed by CD4 CTL, including CX3CR1 (59, 73), perforin (74), and GrK (75). The exact timing and sequence of induction of these molecules remains to be investigated, which can provide further clues to the spatial location of their differentiation. Rapidly evolving methods for fate mapping, lineage tracing or cell tracking in murine models are also of interest to answer this question.

Even though it seems reasonable that CD4 CTL acquire their cytotoxic properties at the site of inflammation, circulating CD4 CTL might not simply consist of locally formed CD4 CTL returning to the circulation as effector memory T cells upon resolving local inflammation. CMV-specific T cells for instance are predominantly found in the circulation, scanning the endothelial cells lining the blood vessels, as these are a critical site for CMV replication and latency (1, 76). Interestingly, the re-expression of CD45RA on circulating CD4 CTL might identify subsets of CD4 CTL that have developed elsewhere and have returned to the peripheral circulation. It has been proposed that re-expression of CD45RA indicates quite some time has passed since the last contact with the cognate antigen (77). Additionally, differentiated CD4^+^ T cells become more responsive to antigen-independent bystander activation through various inflammatory cytokines such as IL-1β, IL-2, and IL-12 (31). One might therefore speculate that CD4 CTL re-expressing CD45RA are a subset of cytotoxic cells going rogue; that they have lost track of their cognate antigen and are now inflicting damage nonspecifically upon bystander activation. Additionally, given that IL-12 signaling is also implicated in activation of Eomes (44), expression of Eomes in CD4 CTL might be an indication that these cells were activated through bystander activation rather than antigenic stimulation. While this might partly explain phenotypic differences between Eomes^+^ and Eomes^-^ CD4 CTL, the ability of IL-12 to induce Eomes expression in CD4^+^ T cells in absence of TCR signaling remains to be confirmed.

## Conclusion

**Figure 1:**
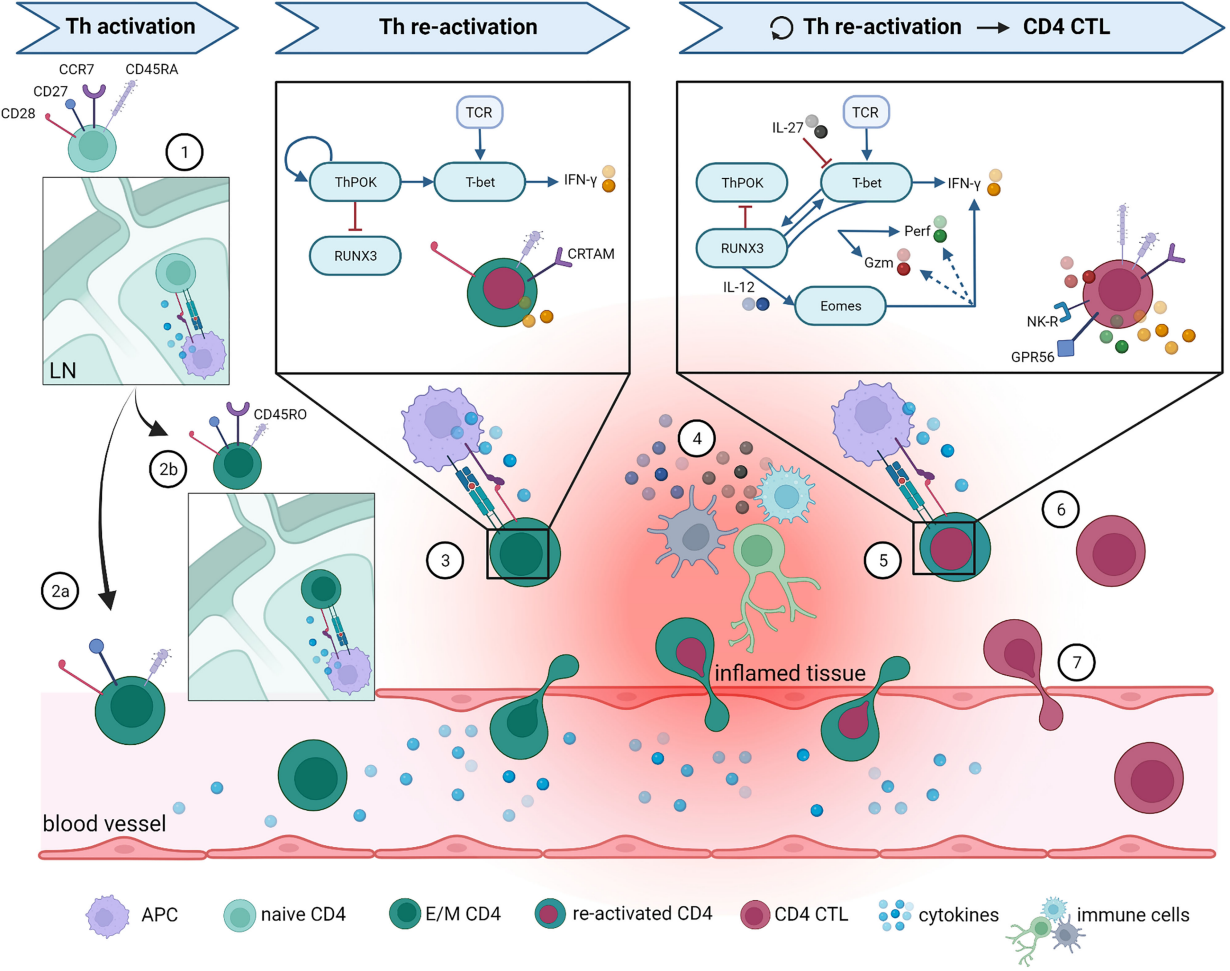
Overview of proposed CD4 CTL development pathway and phenotype. The most important steps in Th and CD4 CTL differentiation are illustrated here. (1) Naïve CD4^+^ T cells enter lymph nodes (LN) and are activated upon binding to their cognate antigen. Following this activations, naïve cells either differentiate into (2a) effector Th cells that can directly migrate towards the site of inflammation, or into (2b) central memory Th cells that can quickly expand upon repeated infection. After re-activation in LN, central memory Th cells can also differentiate into effector Th cells and migrate towards inflamed tissue. (3) At the site of inflammation, effector CD4^+^ Th cells are re-activated by antigen-presenting cells (APC) and start producing effector molecules. For clarity reasons, only the pathway activated in Th1-skewed cells is specified here, as it appears that Th1 skewing is involved in CD4 CTL development. (4) Infiltrated CD4^+^ T cells can alternatively be activated non-specifically through cytokines produced by other activated local immune cells, a process termed bystander activation. Surviving Th memory cells return to the circulation once the inflammation is resolved. (5) When the inflammation persists or returns (for instance in case of a latent viral infection), memory Th cells are re-activated on multiple occasions, which ultimately leads to CD4 CTL development. Upon gaining their cytotoxic potential, CD4 CTL either (6) remain present at the local tissue, or (7) re-enter the circulation. What guides their fate after acquisition of cytotoxic features is currently unknown. More details on the molecules represented in this figure and the accompanying references are given in the main text. Remaining abbreviations: TCR = T cell receptor; Gzm = granzymes; NK-R = NK receptors. Figure created with BioRender.com.

In **Figure 1**, an overview is given of the most robust markers defining CD4 CTL as discussed in this review, as well as the pathways thought to be involved in CD4 CTL development. Although some markers are known to be shared across CD4 CTL, recent insights from single cell multi-omics studies demonstrate that CD4 CTL are a heterogeneous population of T cells, albeit small in absolute cell numbers (depending on age and health status of the donor). Results from a study analyzing donors from different backgrounds suggested that the nature and type of infection leading to CD4 CTL formation shapes the molecular profile of the resulting CD4 CTL (51). This explains why some authors conclude that CD4 CTL are derived from Th1 (40) and others that CD4 CTL can arise from different lineages (42). The unprecedented potential of single cell analysis techniques to detect rare cell populations will undoubtedly lead to discovery of CD4 CTL in other pathologies, as was recently demonstrated for COVID-19 (78), HIV (79), Parkinson’s disease (80), Sjögren’s disease (81), colitis (82), colorectal cancer (83), and cutaneous T cell lymphoma (84). Further mechanistic studies based on these findings will also lead to more insight in the cell lineage(s) that CD4 CTL develop from.

Apart from their origin, there is still much left to be learned about CD4 CTL at a functional level. The unique properties of CD4 CTL compared to CD8^+^ effector T cells are underexposed, since to date most studies comparing both cell types have focused on their similarities. The kinetics of the activation of CD4 CTL versus CD8^+^ effector T cells is one example where both cell types appear to differ. Recently it has been shown that the mechanism of CD4 CTL-mediated killing of tumor cells is largely similar to that of CD8^+^ effector cells, but with delayed kinetics (85). It remains to be confirmed if the kinetics of CD4 CTL triggering in events such as re-activation of persistent viral infections, or autoimmune flare-ups, are similarly delayed compared to CD8^+^ effector activation. Additionally, the impact of CD4 CTL activation on their micro-environment remains an unexplored path. We speculate that after CD4 CTL differentiation still similar clones of CD4^+^ helper T cells exist that originate from a common progenitor effector cell, since CD4 CTL differentiation appears to involve micro-environmental cues as well as a predisposition to become cytotoxic. Thus it seems likely that not all CD4^+^ helper cells specific for the same antigenic epitope will develop into CD4 CTL. This implicates that in the inflamed tissue, some CD4^+^ helper T cells and CD4 CTL will compete for the same antigenic epitope. Given that a proportion of CD4 CTL will degranulate following TCR triggering (73), CD4 CTL might prevent activation of other CD4^+^ T cells through killing of antigen-presenting cells (APC). However, we have shown recently that CD4 CTL are capable of enhancing proliferation and Th17-skewing of CD4^+^ helper T cells in their vicinity (25), which could function as a compensatory mechanism to keep the inflammatory response ongoing. Whether CD4 CTL indeed degranulate following contact with APC, and whether this subsequently induces apoptosis in APC remains to be confirmed experimentally. If so, CD4 CTL might paradoxically also contribute to the declining responsiveness of the immune system commonly observed in aging.

If CD4 CTL can be divided further into different subsets, each with their own function and characteristics, defining the exact molecular mechanism of the conversion from CD4 helper to CD4 CTL will be challenging. Recent developments in analysis of multidimensional high-parameter datasets provide new opportunities to track development of CD4 CTL, using software packages like Infinicyt (86, 87) or Wishbone (88). Although experimentally challenging, the heterogeneity of CD4 CTL can be turned into an advantage if this would allow for specific subtypes of CD4 CTL to be targeted therapeutically. Such personalized therapies could spare the potentially useful CD4 CTL that for instance are involved in tumor immunity and infectious disease, and thereby minimize possible detrimental side effects as only the damaging subset is affected.

## Author Contributions

CH and GD wrote the text. CH designed the figure. NH and BB critically revised the manuscript. All authors have read and approved the submitted version of the manuscript.

## Funding

CH received funds from Stichting MS Research. GD received funds from Charcot Stichting. NH received funds from FWO and Charcot Stichting. BB received funds from Charcot Stichting, MSIF, MoveS, and Stichting MS Research.

## Conflict of Interest

The authors declare that the research was conducted in the absence of any commercial or financial relationships that could be construed as a potential conflict of interest.

## Publisher’s Note

All claims expressed in this article are solely those of the authors and do not necessarily represent those of their affiliated organizations, or those of the publisher, the editors and the reviewers. Any product that may be evaluated in this article, or claim that may be made by its manufacturer, is not guaranteed or endorsed by the publisher.
